# Joy

**Published:** 1889-10-26

**Authors:** 


### Words of Consolation.

### JOY.

*(For Reading to the Sick.)*

People are too apt to think that religion is gloomy, and that the practice of it would make their lives cheerless and sad; and, unfortunately, many true Christians by their moroseness and uncharitable remarks on their neighbours' shortcomings give some colour to the accusations. Again, when a man sees, after years of carelessness, the awful wickedness of sin, he cannot but grieve and feel despondent that he should himself have fallen so often from the path of duty to his Creator and his Lord. It seems to him that the best of his life should be spent in sorrow and penitence. But our blessed Lord, who came on earth to preach the gospel of glad tidings to the poor, and to heal the broken-hearted, does not wish us to lead miserable lives. "Sorrowful, yet always rejoicing," is the very watchword of the Christian. Joy is in the front of our Saviour's teaching; His disciples were to rejoice and be exceeding glad when they were persecuted for His name's sake, and before His passion He tells them "their sorrow should be turned into joy," and their "joy no man taketh from them." After our Lord's ascension He sent His Holy Spirit to comfort and strengthen their hearts, and it is the fruit of this Divine Comforter, joy, which is the sustaining power of our lives, for the word comfort does not only mean to soothe and console, but also to strengthen and invigorate.

"The joy of the Lord is your strength" was the encouragement given by Nehemiah to the Jews on their return from captivity, and this strength may be ours also. It is God's will that we should be joyful, and we cannot fail to be so if the Holy Spirit dwells in our hearts. St. Paul's teaching is ever to the same point, "Rejoice in the Lord alway, and again I say rejoice," sounds like a trumpet call throughout the whole Church. "Rejoice evermore," he says to the Thessalonians, but adds to the charge "pray without ceasing," that being the means by which we may secure the blessing of a cheerful heart to ourselves.

Our blessed Saviour sets us an example in His own person. We can picture Him "rejoicing with them that do rejoice" at the wedding of Cana in Galilee, where we may surely think of Him, not sitting apart in moody silence, but as enjoying the bright happiness and innocent merriment of that bridal feast.

Joy or pleasure in our work brings it to perfection; we have such energy and interest in it, that we try to do our very best, and in general succeed, according to the old saying, "The excellence of the work is in proportion to the joy of the workman." This gift of the Holy Ghost is an immense spiritual force. It is the "oil of gladness" dropping down from on high, making the Christian's life work smoothly and evenly, and in perfect order. "Joy is necessary to the perfection of our being,' says a pious bishop, "and that of every created thing."

